# Common and distinct functional stability abnormalities across three major psychiatric disorders

**DOI:** 10.1016/j.nicl.2020.102352

**Published:** 2020-07-17

**Authors:** Jiajia Zhu, Shujun Zhang, Huanhuan Cai, Chunli Wang, Yongqiang Yu

**Affiliations:** a Department of Radiology, The First Affiliated Hospital of Anhui Medical University, Hefei 230022, China; b Department of Clinical Laboratory, The First Affiliated Hospital of Anhui Medical University, Hefei 230022, China

**Keywords:** Functional stability, Dynamic functional connectivity, Functional MRI, Resting state, Psychiatric disorders

## Abstract

Delineating the neuropathological characteristics across psychiatric disorders is critical for understanding their pathophysiology. The purpose of this study was to investigate common and distinct brain functional abnormalities across psychiatric disorders by using functional stability, a recently developed dynamic functional connectivity approach. Resting-state functional magnetic resonance imaging data were collected from a transdisease sample of healthy controls (n = 115) and individuals with schizophrenia (SZ) (n = 47), bipolar disorder (BD) (n = 44), and attention deficit/hyperactivity disorder (ADHD) (n = 40). Functional stability of each voxel was calculated by measuring the concordance of dynamic functional connectivity over time. Differences in functional stability among the four groups were assessed voxel-wisely. Compared to healthy controls, individuals with SZ demonstrated a distributed pattern of higher functional stability in the bilateral inferior temporal gyrus yet lower stability in the bilateral calcarine sulcus and left insula; individuals with BD only manifested local higher stability in the left inferior temporal gyrus; no differences were found between ADHD and healthy individuals. Notably, individuals with SZ and BD had common higher functional stability in the left inferior temporal gyrus, whereas higher functional stability in the right inferior temporal gyrus and lower stability in the bilateral calcarine sulcus and left insula were unique abnormalities in individuals with SZ. Additionally, direct comparisons between disorders revealed that individuals with SZ showed lower functional stability in the right calcarine sulcus compared to those with BD and higher stability in the left inferior temporal gyrus compared to those with ADHD. However, no significant associations between functional stability and clinical symptoms were observed. Our findings suggest that the functional stability approach has the potential to be extended to the domain of psychiatry and encourage further investigations of shared and unique neuropathology of psychiatric disorders.

## Highlights

• Functional stability is a recently developed dynamic functional connectivity approach.
• Schizophrenia individuals had a distributed pattern of higher and lower stability.
• Individuals with bipolar disorder only manifested local higher stability.
• Individuals with attention deficit/hyperactivity disorder exhibited no stability differences.
• Psychiatric disorders show common and distinct functional stability abnormalities.

## 1 Introduction

The current notion that psychiatric disorders are distinct and independent diagnostic categories remains challenging. In clinical practice, psychiatric disorders tend to have heterogeneous clinical presentations with high co-occurrence (Insel et al., 2010, Merikangas et al., 2010, Sanislow et al., 2010). Although mental disorders such as schizophrenia (SZ), bipolar disorder (BD), and attention deficit/hyperactivity disorder (ADHD) display unique and specific symptoms, many of their clinical symptoms are shared by multiple disorders. Moreover, despite common genetic abnormalities across psychiatric disorders (Consortium, 2013, Purcell et al., 2009), the neuropathological characteristics across psychiatric disorders have yet to be completely elucidated. Identifying both commonalities and differences in brain abnormalities among psychiatric disorders will be instrumental for the development of a precision medicine approach in psychiatry, encompassing more precise and objective diagnoses as well as more effective treatments.

Resting-state functional magnetic resonance imaging (fMRI) constitutes a useful paradigm that permits investigators to non-invasively examine intrinsic brain functional connectivity, i.e., the temporal coherence of blood oxygen level-dependent (BOLD) contrast between different brain regions in the absence of a task (Barkhof et al., 2014, Biswal et al., 1995, Fox and Raichle, 2007). Resting-state functional connectivity approaches have been broadly applied to the domain of psychiatry and have enjoyed significant success in detecting the neurobiological abnormalities and their associations with clinical manifestations in psychiatric disorders (Chan et al., 2019, Dong et al., 2017, Gratton et al., 2019, Posner et al., 2014, Syan et al., 2018, Vargas et al., 2013, Zhu et al., 2017, Zhuo et al., 2018). A majority of previous functional connectivity studies have traditionally relied on static analytic methods that assume stable patterns of connectivity across time. Nevertheless, the brain is not a static organ, and time-varying profiles of functional connectivity are evident across a broad range of task states and during periods of unconstrained rest (Hutchison et al., 2013, Preti et al., 2017). Therefore, exploiting the dynamical properties of functional connectivity instead of the classical static connectivity could open new avenues to interpret brain functioning at different timescales (Calhoun et al., 2014, Chang and Glover, 2010, Hutchison et al., 2013, Preti et al., 2017). Recently, by providing information that is inaccessible through static connectivity analysis, dynamic functional connectivity approaches hold great promise in further revealing the underlying neuropathology of mental disorders such as SZ (Damaraju et al., 2014, Du et al., 2016, Sakoglu et al., 2010), BD (Nguyen et al., 2017, Pang et al., 2018, Wang et al., 2020, Wang et al., 2019), and ADHD (Cai et al., 2018, Kaboodvand et al., 2020, Wang et al., 2018). While functional connectivity abnormalities identified by the static and dynamic analytic methods have the potential to differentiate pathophysiological characteristics between psychiatric disorders, the considerable heterogeneity both in effect sizes and regional distribution of functional connectivity abnormalities across studies has limited the conclusions that have been drawn to date.

Although the brain must dynamically integrate, coordinate, and respond to internal and external stimuli, maintaining stable and consistent representation of information by distributed neural activity and connectivity patterns over time is also essential for consciousness (Dehaene et al., 2017). From a static perspective, prior studies have examined stability (often termed as reliability) of resting-state functional connectivity between multiple fMRI sessions (Mueller et al., 2015, Noble et al., 2017a, Noble et al., 2017b, Shehzad et al., 2009). From a dynamic perspective, a more recent work has characterized stability of the brain’s functional architecture in a voxel-wise manner by measuring the concordance of dynamic functional connectivity over time (Li et al., 2019). In that study, Li and colleagues delineated a profile of the brain’s stability property, characterized by a distribution pattern of higher stability in high-order association regions and lower stability in unimodal regions along with its modifiability by different mental states. This newly developed functional stability approach may hold the potential to serve as a research tool for identifying candidate imaging biomarkers and draw a more refined picture of the neural mechanisms of psychiatric disorders.

In this study, we applied the functional stability approach to resting-state fMRI data from a cross-disorder sample of healthy controls and individuals with SZ, BD, and ADHD. Our objective was to explore functional stability abnormalities in these psychiatric populations. We hypothesized that different psychiatric disorders would show common and distinct functional stability abnormalities.

## 2 Materials and methods

### 2.1 Participants

The participants were from the Consortium for Neuropsychiatric Phenomics (CNP) (Poldrack et al., 2016), a publicly-available dataset. The CNP dataset is available via the OpenfMRI project (http://openfmri.org) (Poldrack et al., 2013) and consists of demographic, clinical and neuroimaging data from a large sample of right-handed adults aged 21–50 years: healthy controls (n = 130) and individuals with SZ (n = 50), BD (n = 49), and ADHD (n = 43). When comparing controls and patients with multiple psychiatric disorders, matched demographic data can be achieved by selecting healthy controls for each group of psychiatric disorders. However, this may result in selection biases. To reduce the selection biases, we used all healthy controls in this study. Diagnoses were determined using the Structured Clinical Interview for DSM-IV (SCID) supplemented by the Adult ADHD Interview. Full details regarding the participants have been described in the data descriptor publication (Poldrack et al., 2016). Out of all participants, 12 had missing imaging data, 13 had excessive head motion (i.e., defined *a priori* as translational or rotational motion parameters > 3 mm or 3° during fMRI scanning), and 1 had errors in the spatial normalization step during fMRI preprocessing. These participants were excluded from subsequent analyses. The demographic information of the final sample is shown in Table 1.

**Table 1:** Demographic characteristics of the sample.

Characteristics	HC	SZ	BD	ADHD	Statistics	*P*
Number of subjects	115	47	44	40		
Gender (female/male)	53/62	12/35	19/25	19/21	*χ^2^* = 6.55	0.088^a^
Age (years)	31.1 ± 8.6	36.5 ± 8.8	35.0 ± 9.1	32.1 ± 10.4	*F* = 4.96	0.002^b^
Education (years)	15.1 ± 1.7	12.6 ± 1.8	14.7 ± 2.0	14.7 ± 1.8	*F* = 22.55	< 0.001^b^
FD (mm)	0.17 ± 0.09	0.26 ± 0.17	0.18 ± 0.09	0.18 ± 0.12	*F* = 7.34	< 0.001^b^

The data are presented as the mean ± standard deviation. Abbreviations: HC, healthy controls; SZ, schizophrenia; BD, bipolar disorder; ADHD, attention deficit/hyperactivity disorder; FD, frame-wise displacement.

a The *P* value was obtained by Pearson Chi-square test.

b The *P* value was obtained by one-way ANOVA.

Clinical symptoms were assessed for individuals with psychiatric disorders. Specifically, severity of psychotic symptoms was evaluated using the Scale for the Assessment of Positive Symptoms (SAPS) (Andreasen, 1984b) and the Scale for the Assessment of Negative Symptoms (SANS) (Andreasen, 1984a). Subscale scores for each symptom dimension were calculated, with the SAPS measuring positive symptoms including hallucinations, delusions, bizarre behavior, and thought disorder and the SANS measuring negative symptoms including affective flattening, alogia, avolition/apathy, anhedonia/asociality, and attention. The twenty-eight item Hamilton Rating Scale for Depression (HAMD) was applied to capture severity of depressive symptoms (Hamilton, 1960). The eleven item Young Mania Rating Scale (YMRS) was utilized to assess severity of manic symptoms (Young et al., 1978). Of note, the SAPS and SANS scores were available for individuals with SZ and BD; the HAMD and YMRS scores were applicable for individuals with SZ, BD, and ADHD.

### 2.2 Imaging acquisition

MRI data were obtained using the 3.0-Tesla Siemens Trio scanner. High-resolution structural MPRAGE images were acquired with the following parameters: repetition time (TR) = 1900 ms; echo time (TE) = 2.26 ms; field of view (FOV) = 250 mm × 250 mm; matrix = 256 × 256; slice thickness = 1 mm, no gap; and 176 sagittal slices. Resting-state BOLD fMRI data were collected using a T2*-weighted echo planar imaging (EPI) sequence with the following parameters: TR = 2000 ms; TE = 30 ms; flip angle = 90°; FOV = 192 mm × 192 mm; matrix = 64 × 64; slice thickness = 4 mm; 34 axial slices; and 152 time points. All MR images were visually inspected to ensure that only images without visible artifacts, lesions, and regional deformations were included in subsequent analyses.

### 2.3 fMRI data preprocessing

Resting-state BOLD data were preprocessed using Statistical Parametric Mapping software (SPM12, http://www.fil.ion.ucl.ac.uk/spm) and Data Processing & Analysis for Brain Imaging (DPABI, http://rfmri.org/dpabi) (Yan et al., 2016). The first 5 volumes for each participant were discarded to allow the signal to reach equilibrium and the participants to adapt to the scanning noise. The remaining volumes were corrected for the acquisition time delay between slices. Then, realignment was performed to correct the motion between time points. Head motion parameters were computed by estimating the translation in each direction and the angular rotation on each axis for each volume. All BOLD data of the final sample were within the defined motion thresholds (i.e., translational or rotational motion parameters<3 mm or 3°). We also calculated frame-wise displacement (FD), which indexes the volume-to-volume changes in head position (Table 1). Several nuisance covariates (the linear drift, the estimated motion parameters based on the Friston-24 model, the spike volumes with FD > 0.5, the white matter signal, and the cerebrospinal fluid signal) were regressed out from the data. The datasets were then band-pass filtered using a frequency range of 0.01 to 0.1 Hz. In the normalization step, individual structural images were firstly co-registered with the mean functional image; then the transformed structural images were segmented and normalized to the Montreal Neurological Institute (MNI) space using a high-level nonlinear warping algorithm, that is, the diffeomorphic anatomical registration through the exponentiated Lie algebra (DARTEL) technique (Ashburner, 2007). Finally, each filtered functional volume was spatially normalized to MNI space using the deformation parameters estimated during the above step and resampled into a 3-mm cubic voxel.

### 2.4 Functional stability calculation

**Fig. 1:**
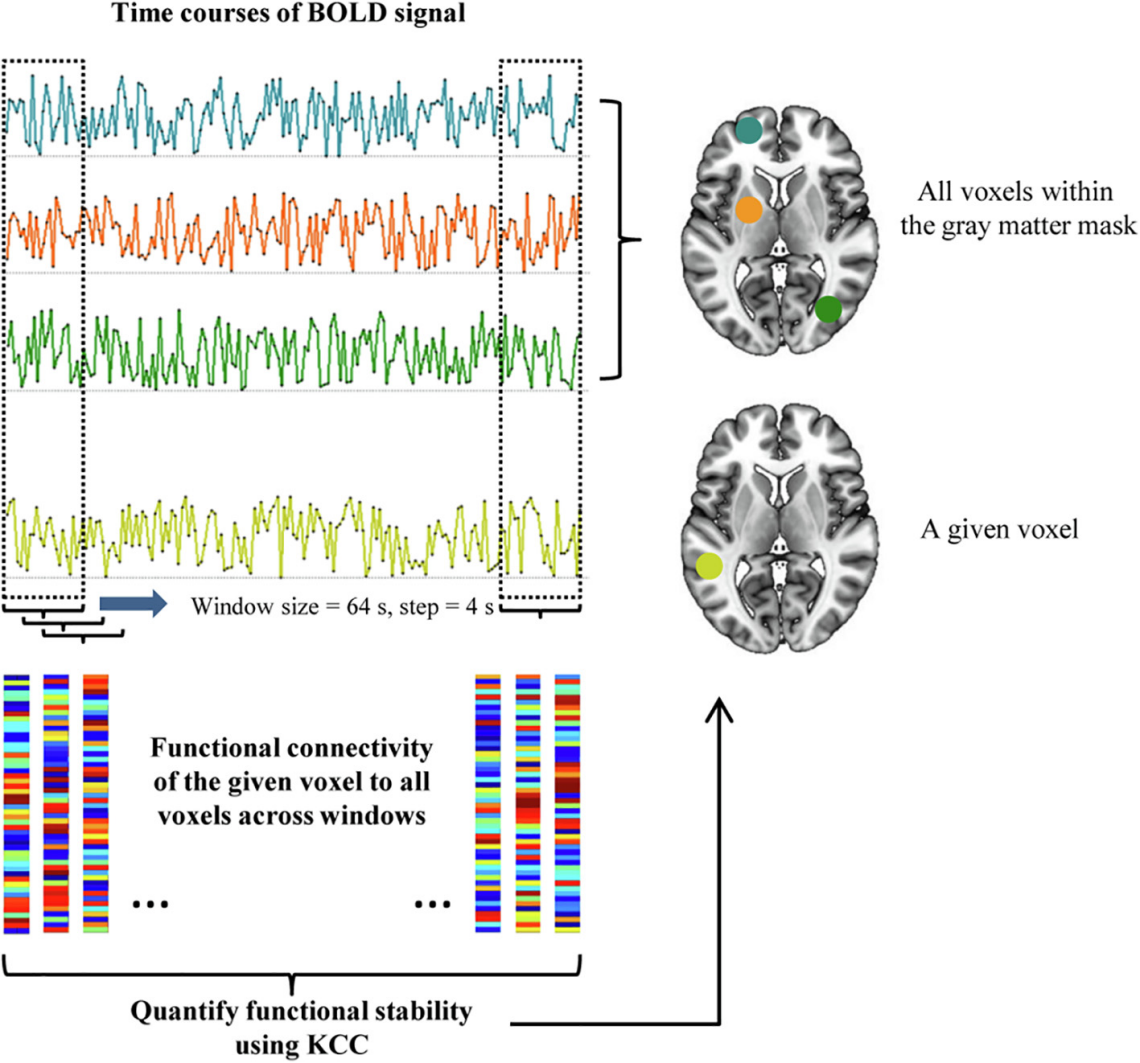
Schematic representation of the functional stability calculation. Firstly, dynamic functional connectivity analysis was performed using a sliding-window approach, with the window size being 64 s and the sliding step being 4 s. For a given voxel, we calculated Pearson’s correlation coefficients between its time course and those of all other voxels within the gray matter mask, resulting in a series of dynamic functional connectivity maps across time windows for that voxel. Then, functional stability of that voxel was quantified by using KCC of these dynamic functional connectivity maps with time windows as raters. Abbreviations: BOLD, blood oxygen level-dependent; KCC, Kendall’s concordance coefficient.

According to a recently published study (Li et al., 2019), the calculation procedure of functional stability is summarized in Fig. 1. Firstly, dynamic functional connectivity analysis was performed using a sliding-window approach (Hutchison et al., 2013), with the window size being 64 s and the sliding step being 4 s (Li et al., 2019). For a given voxel j, we calculated Pearson’s correlation coefficients between its time course and those of all other voxels within the gray matter mask, resulting in a series of dynamic functional connectivity maps across time windows for voxel j. Then, functional stability of voxel j was quantified by using Kendall’s concordance coefficient (KCC) of these dynamic functional connectivity maps with time windows as raters based on the following equation:

$$KCC=\frac{\sum_{n=1}^{N}R_{n}^{2}-\frac{1}{N}{\left(\sum_{n=1}^{N}R_{n}\right)}^{2}}{{\frac{1}{12}K}^{2}\left(N^{3}-N\right)}$$

where K is the number of time windows, N is the number of connections of voxel j with all other voxels within the gray matter mask, and R_n_ is the sum of rank for the n-th connection across all windows. Specifically, all participants had the same number of time windows, resulting in the same K. The gray matter mask used to confine analyses in this study was created by thresholding the mean gray matter density map across participants at 0.2 and intersected with a group-level mask of 90% coverage of all functional images. Thus, N was equal to the number of all voxels within the mask minus one, which was also same for all participants. For the n-th connection (n = 1, 2, ⋯, N), R_n_ was defined as the sum of rank across all the K windows, i.e., R_n_ = R_n_(1) + R_n_(2) + ⋯ + R_n_(K). For each window, connections were ranked on the basis of their functional connectivity strength. To ensure that results were not biased by different numbers of connections, we did not apply any thresholding and a fully connected matrix consisting of both positive and negative connections was used for ranking, with the sign of negative connections rather than their absolute values considered in the ranking. After the resultant functional stability maps were derived, they were further standardized into z-scores by subtracting the mean and dividing by the standard deviation of global values within the gray matter mask, so that they could be averaged and compared across subjects. Finally, these stability maps were spatially smoothed with a Gaussian kernel of 6 mm × 6 mm × 6 mm full-width at half maximum (FWHM). For a voxel or region, a higher stability value (KCC) means that its dynamic functional architecture configuration is more consistent and stable over time, and a lower stability value reflects its ability to frequently and rapidly shift from one brain state to another.

### 2.5 Statistical analysis

Group differences in functional stability among the four groups were tested using a voxel-wise one-way analysis of covariance (ANCOVA) with age, gender, education, and FD as nuisance covariates followed by *post-hoc* two-sample *t*-tests on paired groups. The *post-hoc* pairwise comparisons were conducted within a mask exhibiting functional stability differences from the ANCOVA (*P* < 0.05, uncorrected). For the *post-hoc* pairwise analyses, multiple comparisons were corrected using the cluster-level family-wise error (FWE) method, resulting in a cluster defining threshold of *P* = 0.001 and a corrected cluster significance of *P* < 0.05, following current standard (Eklund et al., 2016). Significant voxels were labeled as different regions with reference to the automated anatomical labeling (AAL) atlas, with the minimum percentage of voxels covering an atlas region being 70%. If significant inter-group differences in functional stability were identified, mean functional stability values of each significant cluster (i.e., a set of contiguous voxels) were extracted and used for region of interest (ROI)-based analyses. The associations between the extracted functional stability values and clinical symptoms (i.e., the SAPS, SANS, HAMD, and YMRS scores) were examined using partial correlations with age, gender, education, and FD as nuisance covariates. Multiple comparison correction for the correlation analyses was performed using the false discovery rate (FDR) method, with a corrected *P* < 0.05. To test the specificity of associations with symptoms, we selected the left amygdala and left thalamus from the AAL atlas as control ROIs to perform a control analysis, because these two regions are frequently reported to show functional abnormalities in psychiatric disorders in previous literature.

### 2.6 Validation analysis

The following procedures were conducted to further evaluate the reproducibility of our findings. First, to determine whether our main results depended on the choice of different sliding windows, we calculated functional stability using three other combinations of window size and sliding step (window size = 50 s and sliding step = 4 s; window size = 80 s and sliding step = 4 s; window size = 64 s and sliding step = 2 s) and then repeated the group comparison analyses. Second, considering that spatial smoothing may influence the results, we used two other Gaussian kernels of 4 mm and 8 mm FWHM to smooth functional stability maps and then reran the voxel-wise group comparison analyses. Third, given that global signal regression (GSR) is still a controversial topic in resting-state fMRI analyses (Murphy and Fox, 2016, Xu et al., 2018), we also repeated our functional stability analyses using fMRI data with GSR instead of white matter and cerebrospinal fluid signal regression. Fourth, the functional stability measurement described above was conducted in a voxel-to-voxel manner, thus incurring a large computational load. To reduce the computational load, we also used a voxel-to-network approach, in which functional stability of a given voxel was computed as KCC of dynamic functional connectivity of that voxel to 7 networks that are widely used in the resting-state fMRI literature (Yeo et al., 2011). Finally, we did not perform smoothing during preprocessing of fMRI data, but rather smoothed functional stability maps before statistical analysis. To test whether different smoothing procedures influenced the results, we also performed smoothing during preprocessing of fMRI data and then calculated functional stability maps that were directly used for statistical analysis.

## 3 Results

### 3.1 Spatial distribution of functional stability

**Fig. 2:**
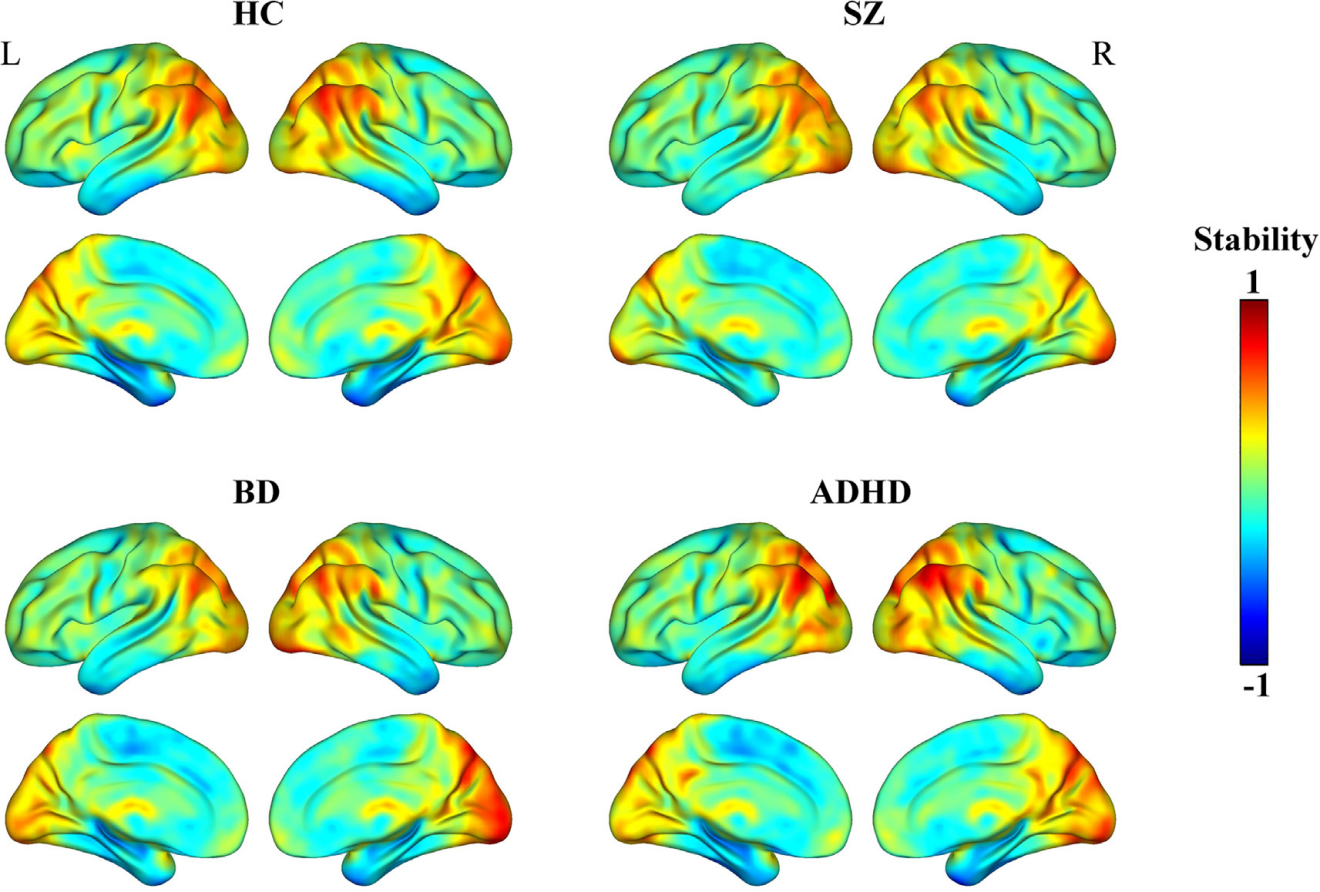
Spatial distribution maps of functional stability. The functional stability maps are averaged across subjects within groups. Abbreviations: HC, healthy controls; SZ, schizophrenia; BD, bipolar disorder; ADHD, attention deficit/hyperactivity disorder; L, left; R, right.

The four groups exhibited similar spatial distribution of functional stability (Fig. 2). Specifically, brain regions with higher functional stability were mainly located in the lateral prefrontal cortex, orbitofrontal cortex, superior parietal lobule, intraparietal sulcus, angular gyrus, supramarginal gyrus, posterior cingulate cortex/precuneus, lateral occipital cortex, cuneus, calcarine sulcus, lingual gyrus, posterior portion of lateral temporal cortex, and thalamus. By contrast, brain regions with lower functional stability were primarily distributed in the dorsomedial prefrontal cortex, paracentral lobule, anterior and middle parts of lateral temporal cortex, and medial temporal cortex.

### 3.2 Functional stability abnormalities in individuals with SZ, BD, and ADHD

**Fig. 3:**
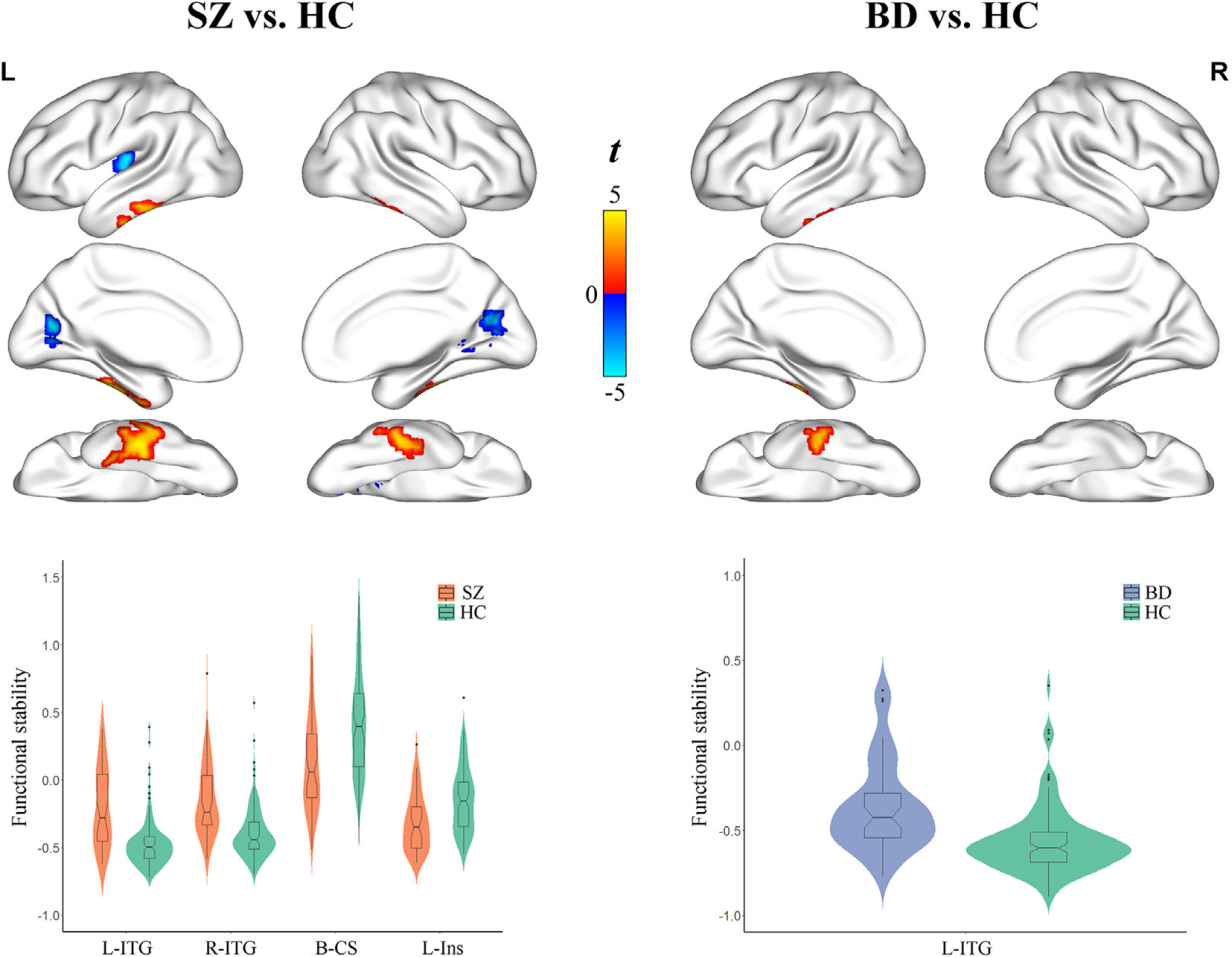
Functional stability abnormalities in individuals with SZ and BD. A combination of violin and box plots shows the distribution and between-group differences of functional stability in the significant clusters. Abbreviations: HC, healthy controls; SZ, schizophrenia; BD, bipolar disorder; L, left; R, right; ITG, inferior temporal gyrus; CS, calcarine sulcus; Ins, insula.

Compared with healthy controls, individuals with SZ exhibited higher functional stability in the bilateral inferior temporal gyrus (left, SZ: −0.20 ± 0.296, controls: −0.47 ± 0.183, *P* < 0.001; right, SZ: −0.14 ± 0.278, controls: −0.39 ± 0.197, *P* < 0.001), and lower stability in the bilateral calcarine sulcus (SZ: 0.12 ± 0.351, controls: 0.41 ± 0.381, *P* < 0.001) and left insula (SZ: −0.32 ± 0.210, controls: −0.15 ± 0.240, *P* < 0.001) after controlling for age, gender, education, and FD (*P* < 0.05, cluster-level FWE corrected) (Fig. 3 and Table 2). Relative to controls, individuals with BD showed higher functional stability in the left inferior temporal gyrus (BD: −0.37 ± 0.263, controls: −0.56 ± 0.197, *P* < 0.001) (*P* < 0.05, cluster-level FWE corrected) (Fig. 3 and Table 2). However, there were no significant differences in functional stability between individuals with ADHD and healthy controls (*P* > 0.05, cluster-level FWE corrected). Notably, individuals with SZ and BD had common higher functional stability in the left inferior temporal gyrus, whereas higher functional stability in the right inferior temporal gyrus and lower stability in the bilateral calcarine sulcus and left insula were unique abnormalities in individuals with SZ.

**Table 2:** Brain regions showing functional stability differences between groups.

Regions	Cluster size (voxels)	Peak *t* values	Coordinates in MNI (x, y, z)
SZ > HC			
Left inferior temporal gyrus	238	4.9	−48, −27, −24
**Right inferior temporal gyrus**	**87**	**4.4**	**48, –33, −27**
SZ < HC			
Bilateral calcarine sulcus	203	−4.7	−12, −69, 15
Left insula	53	−5.0	–33, −18, 9
BD > HC			
Left inferior temporal gyrus	64	4.5	−45, −12, −27
SZ < BD			
Right calcarine sulcus	57	−3.9	15, −78, 12
SZ > ADHD			
Left inferior temporal gyrus	76	4.1	−48, −27, −24

Abbreviations: HC, healthy controls; SZ, schizophrenia; BD, bipolar disorder; ADHD, attention deficit/hyperactivity disorder; MNI, Montreal Neurological Institute.

### 3.3 Differences in functional stability between individuals with SZ, BD, and ADHD

**Fig. 4:**
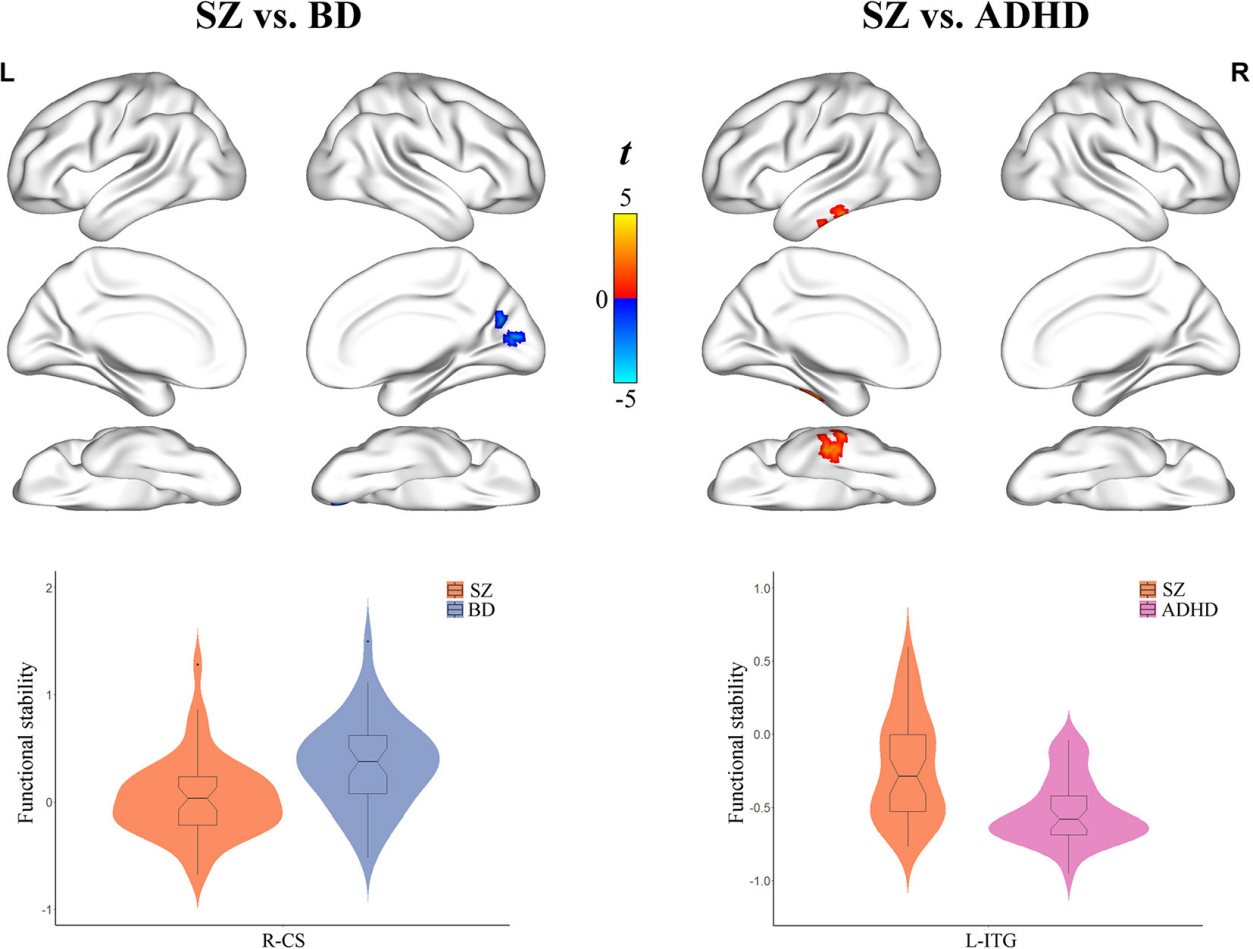
Differences in functional stability between individuals with SZ, BD, and ADHD. A combination of violin and box plots shows the distribution and between-group differences of functional stability in the significant clusters. Abbreviations: SZ, schizophrenia; BD, bipolar disorder; ADHD, attention deficit/hyperactivity disorder; L, left; R, right; CS, calcarine sulcus; ITG, inferior temporal gyrus.

Individuals with SZ had lower functional stability in the right calcarine sulcus (SZ: 0.04 ± 0.366, BD: 0.36 ± 0.409, *P* < 0.001) compared to those with BD after controlling for age, gender, education, and FD (*P* < 0.05, cluster-level FWE corrected) (Fig. 4 and Table 2). Moreover, individuals with SZ exhibited higher functional stability in the left inferior temporal gyrus (SZ: −0.23 ± 0.356, ADHD: −0.53 ± 0.220, *P* < 0.001) relative to those with ADHD (*P* < 0.05, cluster-level FWE corrected) (Fig. 4 and Table 2). However, no significant differences in functional stability were observed between individuals with BD and ADHD (*P* > 0.05, cluster-level FWE corrected).

### 3.4 Associations between functional stability and clinical symptoms

The ROI-based correlations between functional stability and clinical symptoms (i.e., the SAPS, SANS, HAMD, and YMRS scores) in individuals with SZ, BD and ADHD are demonstrated in Tables S1-S3, respectively. Although there were several nominally significant correlations, they did not survive the FDR correction for multiple comparisons (*P* > 0.05, FDR corrected). In addition, correlation analyses were performed in all psychiatric individuals. As shown in Table S4, we also did not find any significant correlations (*P* > 0.05, FDR corrected).

### 3.5 Validation analysis

First, as demonstrated in Fig. S1-S3, our main findings were reproduced when using functional stability derived from three other combinations of window size and sliding step, suggesting that different sliding windows did not significantly influence our functional stability analyses. Second, when using 4 mm and 8 mm FWHM Gaussian kernels to smooth functional stability maps, we found that the voxel-wise group comparison results were similar to those using a 6 mm FWHM Gaussian kernel (Fig. S4 and S5). Third, the results of inter-group comparisons in functional stability using fMRI data with GSR instead of white matter and cerebrospinal fluid signal regression were consistent with the main results without GSR (Fig. S6). Fourth, when using a voxel-to-network approach, we found that most of the group differences in functional stability did not survive the cluster-level FWE correction for multiple comparisons. Only the lower functional stability in the calcarine sulcus in SZ vs. BD individuals was preserved (Fig. S7). This finding may indicate that the voxel-to-voxel stability approach might be more sensitive than the voxel-to-network approach in detecting brain dysfunction in psychiatric disorders. Finally, when using smoothed fMRI data to calculate functional stability, the group differences became nonsignificant with the exception of lower functional stability in the calcarine sulcus in SZ vs. BD individuals (Fig. S8), suggesting that different smoothing procedures affected the functional stability analyses to a certain degree.

## 4 Discussion

To our knowledge, this is the first resting-state fMRI study to adopt a recently developed functional stability approach to investigate brain abnormalities in SZ, BD, and ADHD. Compared with healthy controls, individuals with SZ demonstrated a distributed pattern of higher functional stability in the inferior temporal gyrus yet lower stability in the calcarine sulcus and insula; individuals with BD only manifested local higher stability in the inferior temporal gyrus; no differences were found between ADHD and healthy individuals. Additionally, direct comparisons between disorders revealed that individuals with SZ showed lower functional stability in the calcarine sulcus compared to those with BD and higher stability in the inferior temporal gyrus compared to those with ADHD. Our findings may yield insights into nosology and encourage further research on common and unique pathophysiology of psychiatric disorders.

For a voxel or region, a higher stability value means that its dynamic functional architecture configuration is more consistent and stable over time, and a lower stability value reflects its ability to frequently and rapidly shift from one brain state to another. In the current study, we found higher functional stability mainly located in the default mode, executive control and visual networks, and lower stability primarily distributed in the sensorimotor, auditory and limbic networks. Our observations are largely compatible with previous reports of higher stability in high-order association regions and lower stability in unimodal regions from both static and dynamic perspectives (Li et al., 2019, Mueller et al., 2015, Shehzad et al., 2009, Tomasi et al., 2017). Complex cognitive functions require the brain to integrate and coordinate information from multiple modalities over time (Cole et al., 2014, Vidaurre et al., 2017). High-order regions play a key role in the cognitive and conscious processing given their broadly distributed functional connectivity (Cole et al., 2010). It is reasonable to assume that high stability might provide a foundation for high-order regions to process information over a long time scale and thus offer efficient capacity to facilitate rapid response, which may render the brain adaptive to the environment. By contrast, functional instability in low-order brain regions may indicate that their activity or functional connectivity might change frequently. Neural activity of low-order brain regions is driven by both external stimuli and top-down modulation from high-order regions (Macaluso and Driver, 2005). One may speculate that low-order regions respond to the always-changing world, and thus need to reorganize their functional connectivity with other regions rapidly. The low stability may reflect short time scale to process and accumulate information, which provides a basis for low-order regions to frequently and rapidly reorganize their functional connectivity (Lerner et al., 2011). Despite these findings, future studies are warranted to further elucidate the physiological significance of functional stability. Note that the basic idea behind the functional stability method seems to be comparable to the regional homogeneity (ReHo) approach (Zang et al., 2004). The ReHo is a static fMRI approach that measures the degree of local functional synchronization by calculating the concordance of time course of a given voxel with those of its nearest neighbors. By contrast, the functional stability is a dynamic fMRI method that measures the brain’s functional architecture stability by computing the concordance of dynamic functional connectivity over time. Thus, these two measures characterize different brain functional properties from distinct perspectives (static vs. dynamic and local vs. global).

SZ patients were found to exhibit higher functional stability in the inferior temporal gyrus and lower stability in the calcarine sulcus and insula. Abnormal functional stability increase may limit the ability to rapidly shift from one brain state to another and aberrant stability reduction may cause an inability to maintain a consistent functional organization with other brain regions, which both contribute to the pathophysiology of SZ. The inferior temporal gyrus is implicated in visual perception (Conway, 2018) and multimodal sensory integration (Mesulam, 1998). While some structural MRI studies have revealed volumetric atrophy of the inferior temporal gyrus in SZ patients (Mennigen et al., 2019, Onitsuka et al., 2004), there is empirical evidence from functional MRI data pointing to greater function of this region in SZ, such as higher cerebral blood flow, local neural activity, and functional connectivity (Xu et al., 2015, Zhu et al., 2015, Zhu et al., 2017, Zhuo et al., 2017), which accord with the current observation of higher functional stability in the inferior temporal gyrus in SZ. Structural and functional impairments of the visual cortex in SZ have been extensively reported (Chen et al., 2015, Onitsuka et al., 2007, Schultz et al., 2013, Xu et al., 2015, Zhu et al., 2015, Zhu et al., 2017, Zhuo et al., 2017). Our finding of lower functional stability in the primary visual cortex, in conjunction with these previous reports, may explain the deficits of visual processing in schizophrenia (Butler et al., 2001, Butler et al., 2005). Involvement of the insula is a common finding in neuroimaging studies of SZ (Ebisch et al., 2014, Shepherd et al., 2012, Zhu et al., 2018). The insula is a brain structure with extensive connections to widespread cortical areas and limbic system (Cauda et al., 2011). Thus, it is considered a multifaceted construct engaged in disparate cognitive, affective, and regulatory functions, including interoceptive awareness, emotional responses, and empathic processes (Menon and Uddin, 2010). Many deficits observed in SZ involve these functions and may be linked to insula pathology (Wylie and Tregellas, 2010).

In comparison with healthy controls, we found that individuals with SZ and BD had common higher functional stability in the inferior temporal gyrus. Prior genetic research has demonstrated phenotypic and genetic overlap between SZ and BD (Consortium, 2013, Purcell et al., 2009). Our present finding of shared functional stability abnormalities between individuals with SZ and BD may partially reflect genetic overlap. The lack of functional stability differences between individuals with ADHD and controls may imply that the neurobiological mechanism of ADHD cannot be identified by the functional stability approach. Direct comparisons between disorders revealed significant stability differences in the calcarine sulcus between SZ and BD and in the inferior temporal gyrus between SZ and ADHD. Those regions are identical to the regions where differences were identified between individuals with SZ and healthy controls, which may in part endorse the idea that SZ shows more severe brain functional damage than BD and ADHD. Furthermore, functional stability of those regions not only can be utilized to distinguish between SZ patients and healthy controls, but also may serve as potential biomarkers for discriminating SZ subjects from other psychiatric populations. Collectively, our findings of common and distinct brain functional stability abnormalities across psychiatric disorders may contribute to the further development of new diagnostic methods (e.g., on the basis of biological signatures rather than symptomatic indicators) in psychiatry.

Our study has several limitations that must be considered. First, our small sample size limits the statistical power and the generalizability of our findings. Larger sample sizes would be necessary to detect subtle brain abnormalities and potential brain-behavior associations. Second, the four groups were not well matched for demographic variables. However, we minimized this effect by including the unmatched variables as covariates of no interest. Third, our interpretation may be affected by some potential confounders, such as medication use and/or long illness duration in the patient groups. Future studies with drug-naive first-episode patients are required to further validate our findings. Fourth, we focused our analysis on resting-state rather than task-based fMRI data. Although there is evidence that task states can substantially modify functional stability (Li et al., 2019), use of resting-state fMRI may facilitate generalization of our findings since collection of these data is now commonplace in semi-standardized ways across human imaging studies. Fifth, during the resting-state fMRI scans, subjects’ drowsiness or vigilance levels and confounding physiological noise have been shown to influence dynamic functional connectivity (Laumann et al., 2017), which could in turn have consequences for the measurement of functional stability. Future investigations of the brain’s functional dynamics and stability would benefit from recording these physiological variables during scanning to rule out their potential effects. Sixth, our validation analyses suggested that different smoothing procedures including Gaussian kernel parameters and smoothing order affected the results to varying degrees. These findings are of interest and further research is needed to investigate the extent and nature of the effects of smoothing on the functional stability analyses. Finally, the functional stability approach is computationally demanding, i.e., this protocol may take about 1 h for fMRI data preprocessing and 8 h for functional stability calculation for one subject. The large computational load makes it challenging to apply this functional stability technique to routine clinical practice.

In conclusion, we have demonstrated functional stability abnormalities across psychiatric disorders. Briefly, individuals with SZ exhibited a distributed pattern of higher and lower functional stability. In contrast, individuals with BD only showed local higher stability and no differences were found between ADHD and healthy individuals. Our findings suggest that the functional stability approach has the potential to be extended to psychiatry and encourage further investigations of shared and unique neuropathology of psychiatric disorders.

## Funding

The work was supported by the National Natural Science Foundation of China (grant numbers: 81801679 and 81771817).

## CRediT authorship contribution statement

**Jiajia Zhu:** Conceptualization, Formal analysis, Funding acquisition, Investigation, Methodology, Software, Validation, Visualization, Writing - original draft, Writing - review & editing. **Shujun Zhang:** Data curation, Formal analysis, Investigation, Methodology, Software, Writing - original draft. **Huanhuan Cai:** Data curation, Formal analysis, Investigation, Methodology, Software. **Chunli Wang:** Data curation, Formal analysis, Investigation, Methodology, Validation, Visualization. **Yongqiang Yu:** Conceptualization, Formal analysis, Funding acquisition, Project administration, Resources, Supervision, Writing - review & editing.

## Declaration of Competing Interest

The authors declare that they have no known competing financial interests or personal relationships that could have appeared to influence the work reported in this paper.

## References

[b0005] Andreasen N.C. (1984). Scale for the Assessment of Negative Symptoms (SANS).

[b0010] Andreasen N.C. (1984). Scale for the Assessment of Positive Symptoms (SAPS).

[b0015] Ashburner J. (2007). A fast diffeomorphic image registration algorithm. Neuroimage.

[b0020] Barkhof F., Haller S., Rombouts S.A. (2014). Resting-state functional MR imaging: a new window to the brain. Radiology.

[b0025] Biswal B., Yetkin F.Z., Haughton V.M., Hyde J.S. (1995). Functional connectivity in the motor cortex of resting human brain using echo-planar MRI. Magn. Reson. Med..

[b0030] Butler P.D., Schechter I., Zemon V., Schwartz S.G., Greenstein V.C., Gordon J., Schroeder C.E., Javitt D.C. (2001). Dysfunction of early-stage visual processing in schizophrenia. Am. J. Psychiatry..

[b0035] Butler P.D., Zemon V., Schechter I., Saperstein A.M., Hoptman M.J., Lim K.O., Revheim N., Silipo G., Javitt D.C. (2005). Early-stage visual processing and cortical amplification deficits in schizophrenia. Arch. Gen. Psychiatry.

[b0040] Cai W., Chen T., Szegletes L., Supekar K., Menon V. (2018). Aberrant Time-Varying Cross-Network Interactions in Children With Attention-Deficit/Hyperactivity Disorder and the Relation to Attention Deficits. Biol. Psychiatry Cogn. Neurosci. Neuroimaging.

[b0045] Calhoun V.D., Miller R., Pearlson G., Adali T. (2014). The chronnectome: time-varying connectivity networks as the next frontier in fMRI data discovery. Neuron.

[b0050] Cauda F., D'Agata F., Sacco K., Duca S., Geminiani G., Vercelli A. (2011). Functional connectivity of the insula in the resting brain. Neuroimage.

[b0055] Chan N.K., Kim J., Shah P., Brown E.E., Plitman E., Carravaggio F., Iwata Y., Gerretsen P., Graff-Guerrero A. (2019). Resting-state functional connectivity in treatment response and resistance in schizophrenia: A systematic review. Schizophr. Res..

[b0060] Chang C., Glover G.H. (2010). Time-frequency dynamics of resting-state brain connectivity measured with fMRI. Neuroimage.

[b0065] Chen X., Duan M., Xie Q., Lai Y., Dong L., Cao W., Yao D., Luo C. (2015). Functional disconnection between the visual cortex and the sensorimotor cortex suggests a potential mechanism for self-disorder in schizophrenia. Schizophr. Res..

[b0070] Cole M.W., Bassett D.S., Power J.D., Braver T.S., Petersen S.E. (2014). Intrinsic and task-evoked network architectures of the human brain. Neuron.

[b0075] Cole M.W., Pathak S., Schneider W. (2010). Identifying the brain's most globally connected regions. Neuroimage.

[b0080] Consortium, C.-D.G.o.t.P.G., 2013. Identification of risk loci with shared effects on five major psychiatric disorders: a genome-wide analysis. Lancet 381, 1371-1379. 10.1016/S0140-6736(12)62129-1 PMC3714010 23453885

[b0085] Conway B.R. (2018). The Organization and Operation of Inferior Temporal Cortex. Annu. Rev. Vis. Sci..

[b0090] Damaraju E., Allen E.A., Belger A., Ford J.M., McEwen S., Mathalon D.H., Mueller B.A., Pearlson G.D., Potkin S.G., Preda A., Turner J.A., Vaidya J.G., van Erp T.G., Calhoun V.D. (2014). Dynamic functional connectivity analysis reveals transient states of dysconnectivity in schizophrenia. Neuroimage Clin..

[b0095] Dehaene S., Lau H., Kouider S. (2017). What is consciousness, and could machines have it?. Science.

[b0100] Dong, D., Wang, Y., Chang, X., Luo, C., Yao, D., 2017. Dysfunction of Large-Scale Brain Networks in Schizophrenia: A Meta-analysis of Resting-State Functional Connectivity. Schizophr Bull. 10.1093/schbul/sbx034 PMC5767956 28338943

[b0105] Du Y., Pearlson G.D., Yu Q., He H., Lin D., Sui J., Wu L., Calhoun V.D. (2016). Interaction among subsystems within default mode network diminished in schizophrenia patients: A dynamic connectivity approach. Schizophr. Res..

[b0110] Ebisch S.J., Mantini D., Northoff G., Salone A., De Berardis D., Ferri F., Ferro F.M., Di Giannantonio M., Romani G.L., Gallese V. (2014). Altered brain long-range functional interactions underlying the link between aberrant self-experience and self-other relationship in first-episode schizophrenia. Schizophr. Bull..

[b0115] Eklund A., Nichols T.E., Knutsson H. (2016). Cluster failure: Why fMRI inferences for spatial extent have inflated false-positive rates. Proc. Natl. Acad. Sci. U S A.

[b0120] Fox M.D., Raichle M.E. (2007). Spontaneous fluctuations in brain activity observed with functional magnetic resonance imaging. Nat. Rev. Neurosci..

[b0125] Gratton C., Kraus B.T., Greene D.J., Gordon E.M., Laumann T.O., Nelson S.M., Dosenbach N.U.F., Petersen S.E. (2019). Defining Individual-Specific Functional Neuroanatomy for Precision Psychiatry. Biol. Psychiatry.

[b0130] Hamilton M. (1960). A rating scale for depression. J Neurol. Neurosurg. Psychiatry.

[b0135] Hutchison R.M., Womelsdorf T., Allen E.A., Bandettini P.A., Calhoun V.D., Corbetta M., Della Penna S., Duyn J.H., Glover G.H., Gonzalez-Castillo J., Handwerker D.A., Keilholz S., Kiviniemi V., Leopold D.A., de Pasquale F., Sporns O., Walter M., Chang C. (2013). Dynamic functional connectivity: promise, issues, and interpretations. Neuroimage.

[b0140] Insel T., Cuthbert B., Garvey M., Heinssen R., Pine D.S., Quinn K., Sanislow C., Wang P. (2010). Research domain criteria (RDoC): toward a new classification framework for research on mental disorders. Am. J. Psychiatry.

[b0145] Kaboodvand N., Iravani B., Fransson P. (2020). Dynamic synergetic configurations of resting-state networks in ADHD. Neuroimage.

[b0150] Laumann T.O., Snyder A.Z., Mitra A., Gordon E.M., Gratton C., Adeyemo B., Gilmore A.W., Nelson S.M., Berg J.J., Greene D.J., McCarthy J.E., Tagliazucchi E., Laufs H., Schlaggar B.L., Dosenbach N.U.F., Petersen S.E. (2017). On the Stability of BOLD fMRI Correlations. Cereb Cortex.

[b0155] Lerner Y., Honey C.J., Silbert L.J., Hasson U. (2011). Topographic mapping of a hierarchy of temporal receptive windows using a narrated story. J. Neurosci..

[b0160] Li, L., Lu, B., Yan, C.G., 2019. Stability of dynamic functional architecture differs between brain networks and states. Neuroimage, 116230. 10.1016/j.neuroimage.2019.116230 31577959

[b0165] Macaluso E., Driver J. (2005). Multisensory spatial interactions: a window onto functional integration in the human brain. Trends Neurosci..

[b0170] Mennigen E., Jiang W., Calhoun V.D., van Erp T.G.M., Agartz I., Ford J.M., Mueller B.A., Liu J., Turner J.A. (2019). Positive and general psychopathology associated with specific gray matter reductions in inferior temporal regions in patients with schizophrenia. Schizophr. Res..

[b0175] Menon V., Uddin L.Q. (2010). Saliency, switching, attention and control: a network model of insula function. Brain Struct. Funct..

[b0180] Merikangas K.R., He J.P., Burstein M., Swanson S.A., Avenevoli S., Cui L., Benjet C., Georgiades K., Swendsen J. (2010). Lifetime prevalence of mental disorders in U.S. adolescents: results from the National Comorbidity Survey Replication-Adolescent Supplement (NCS-A). J. Am. Acad. Child. Adolesc. Psychiatry.

[b0185] Mesulam M.M. (1998). From sensation to cognition. Brain.

[b0190] Mueller S., Wang D., Fox M.D., Pan R., Lu J., Li K., Sun W., Buckner R.L., Liu H. (2015). Reliability correction for functional connectivity: Theory and implementation. Hum. Brain. Mapp..

[b0195] Murphy K., Fox M.D. (2016). Towards a consensus regarding global signal regression for resting state functional connectivity MRI. Neuroimage.

[b0200] Nguyen T.T., Kovacevic S., Dev S.I., Lu K., Liu T.T., Eyler L.T. (2017). Dynamic functional connectivity in bipolar disorder is associated with executive function and processing speed: A preliminary study. Neuropsychology.

[b0205] Noble S., Scheinost D., Finn E.S., Shen X., Papademetris X., McEwen S.C., Bearden C.E., Addington J., Goodyear B., Cadenhead K.S., Mirzakhanian H., Cornblatt B.A., Olvet D.M., Mathalon D.H., McGlashan T.H., Perkins D.O., Belger A., Seidman L.J., Thermenos H., Tsuang M.T., van Erp T.G.M., Walker E.F., Hamann S., Woods S.W., Cannon T.D., Constable R.T. (2017). Multisite reliability of MR-based functional connectivity. Neuroimage.

[b0210] Noble S., Spann M.N., Tokoglu F., Shen X., Constable R.T., Scheinost D. (2017). Influences on the Test-Retest Reliability of Functional Connectivity MRI and its Relationship with Behavioral Utility. Cereb. Cortex.

[b0215] Onitsuka T., McCarley R.W., Kuroki N., Dickey C.C., Kubicki M., Demeo S.S., Frumin M., Kikinis R., Jolesz F.A., Shenton M.E. (2007). Occipital lobe gray matter volume in male patients with chronic schizophrenia: A quantitative MRI study. Schizophr. Res..

[b0220] Onitsuka T., Shenton M.E., Salisbury D.F., Dickey C.C., Kasai K., Toner S.K., Frumin M., Kikinis R., Jolesz F.A., McCarley R.W. (2004). Middle and inferior temporal gyrus gray matter volume abnormalities in chronic schizophrenia: an MRI study. Am. J. Psychiatry.

[b0225] Pang Y., Chen H., Wang Y., Long Z., He Z., Zhang H., Liao W., Cui Q. (2018). Transdiagnostic and diagnosis-specific dynamic functional connectivity anchored in the right anterior insula in major depressive disorder and bipolar depression. Prog Neuropsychopharmacol. Biol. Psych..

[b0230] Poldrack R.A., Barch D.M., Mitchell J.P., Wager T.D., Wagner A.D., Devlin J.T., Cumba C., Koyejo O., Milham M.P. (2013). Toward open sharing of task-based fMRI data: the OpenfMRI project. Front. Neuroinform..

[b0235] Poldrack R.A., Congdon E., Triplett W., Gorgolewski K.J., Karlsgodt K.H., Mumford J.A., Sabb F.W., Freimer N.B., London E.D., Cannon T.D., Bilder R.M. (2016). A phenome-wide examination of neural and cognitive function. Sci. Data.

[b0240] Posner J., Park C., Wang Z. (2014). Connecting the dots: a review of resting connectivity MRI studies in attention-deficit/hyperactivity disorder. Neuropsychol. Rev..

[b0245] Preti M.G., Bolton T.A., Van De Ville D. (2017). The dynamic functional connectome: State-of-the-art and perspectives. Neuroimage.

[b0250] Purcell S.M., Wray N.R., Stone J.L., Visscher P.M., O'Donovan M.C., Sullivan P.F., Sklar P. (2009). Common polygenic variation contributes to risk of schizophrenia and bipolar disorder. Nature.

[b0255] Sakoglu U., Pearlson G.D., Kiehl K.A., Wang Y.M., Michael A.M., Calhoun V.D. (2010). A method for evaluating dynamic functional network connectivity and task-modulation: application to schizophrenia. MAGMA.

[b0260] Sanislow C.A., Pine D.S., Quinn K.J., Kozak M.J., Garvey M.A., Heinssen R.K., Wang P.S., Cuthbert B.N. (2010). Developing constructs for psychopathology research: research domain criteria. J. Abnorm. Psychol..

[b0265] Schultz C.C., Wagner G., Koch K., Gaser C., Roebel M., Schachtzabel C., Nenadic I., Reichenbach J.R., Sauer H., Schlosser R.G. (2013). The visual cortex in schizophrenia: alterations of gyrification rather than cortical thickness–a combined cortical shape analysis. Brain Struct. Funct..

[b0270] Shehzad Z., Kelly A.M., Reiss P.T., Gee D.G., Gotimer K., Uddin L.Q., Lee S.H., Margulies D.S., Roy A.K., Biswal B.B., Petkova E., Castellanos F.X., Milham M.P. (2009). The resting brain: unconstrained yet reliable. Cereb. Cortex.

[b0275] Shepherd A.M., Matheson S.L., Laurens K.R., Carr V.J., Green M.J. (2012). Systematic meta-analysis of insula volume in schizophrenia. Biol. Psychiat..

[b0280] Syan S.K., Smith M., Frey B.N., Remtulla R., Kapczinski F., Hall G.B.C., Minuzzi L. (2018). Resting-state functional connectivity in individuals with bipolar disorder during clinical remission: a systematic review. J. Psychiatry Neurosci..

[b0285] Tomasi D.G., Shokri-Kojori E., Volkow N.D. (2017). Temporal Evolution of Brain Functional Connectivity Metrics: Could 7 Min of Rest be Enough?. Cereb. Cortex.

[b0290] Vargas C., Lopez-Jaramillo C., Vieta E. (2013). A systematic literature review of resting state network–functional MRI in bipolar disorder. J. Affect. Disord..

[b0295] Vidaurre D., Smith S.M., Woolrich M.W. (2017). Brain network dynamics are hierarchically organized in time. Proc. Natl. Acad. Sci. U S A.

[b0300] Wang J., Wang Y., Huang H., Jia Y., Zheng S., Zhong S., Chen G., Huang L., Huang R. (2020). Abnormal dynamic functional network connectivity in unmedicated bipolar and major depressive disorders based on the triple-network model. Psychol. Med..

[b0305] Wang J., Wang Y., Huang H., Jia Y., Zheng S., Zhong S., Huang L., Huang R. (2019). Abnormal intrinsic brain functional network dynamics in unmedicated depressed bipolar II disorder. J Affect. Disord..

[b0310] Wang X.H., Jiao Y., Li L. (2018). Identifying individuals with attention deficit hyperactivity disorder based on temporal variability of dynamic functional connectivity. Sci. Rep..

[b0315] Wylie K.P., Tregellas J.R. (2010). The role of the insula in schizophrenia. Schizophr. Res..

[b0320] Xu H., Su J., Qin J., Li M., Zeng L.L., Hu D., Shen H. (2018). Impact of global signal regression on characterizing dynamic functional connectivity and brain states. Neuroimage.

[b0325] Xu Y., Zhuo C., Qin W., Zhu J., Yu C. (2015). Altered Spontaneous Brain Activity in Schizophrenia: A Meta-Analysis and a Large-Sample Study. Biomed Res. Int..

[b0330] Yan C.G., Wang X.D., Zuo X.N., Zang Y.F. (2016). DPABI: Data Processing & Analysis for (Resting-State) Brain Imaging. Neuroinformatics.

[b0335] Yeo B.T., Krienen F.M., Sepulcre J., Sabuncu M.R., Lashkari D., Hollinshead M., Roffman J.L., Smoller J.W., Zollei L., Polimeni J.R., Fischl B., Liu H., Buckner R.L. (2011). The organization of the human cerebral cortex estimated by intrinsic functional connectivity. J. Neurophysiol..

[b0340] Young R.C., Biggs J.T., Ziegler V.E., Meyer D.A. (1978). A rating scale for mania: reliability, validity and sensitivity. Br. J. Psych..

[b0345] Zang Y., Jiang T., Lu Y., He Y., Tian L. (2004). Regional homogeneity approach to fMRI data analysis. Neuroimage.

[b0350] Zhu J., Zhu D.M., Qian Y., Li X., Yu Y. (2018). Altered spatial and temporal concordance among intrinsic brain activity measures in schizophrenia. J. Psychiatr. Res..

[b0355] Zhu J., Zhuo C., Qin W., Xu Y., Xu L., Liu X., Yu C. (2015). Altered resting-state cerebral blood flow and its connectivity in schizophrenia. J. Psychiatr. Res..

[b0360] Zhu J., Zhuo C., Xu L., Liu F., Qin W., Yu C. (2017). Altered Coupling Between Resting-State Cerebral Blood Flow and Functional Connectivity in Schizophrenia. Schizophr. Bull..

[b0365] Zhuo C., Wang C., Wang L., Guo X., Xu Q., Liu Y., Zhu J. (2018). Altered resting-state functional connectivity of the cerebellum in schizophrenia. Brain Imaging Behav..

[b0370] Zhuo C., Zhu J., Qin W., Qu H., Ma X., Yu C. (2017). Cerebral blood flow alterations specific to auditory verbal hallucinations in schizophrenia. Br. J. Psychiatry.

