# Supplementary material for: Common and distinct functional stability abnormalities across three major psychiatric disorders
Source: Neuroimage Clin. 2020 Jul 17;27:102352. doi: 10.1016/j.nicl.2020.102352 (PMC7393318; doi:10.1016/j.nicl.2020.102352)
Supplement: Supplementary data 1 [file mmc1.docx]

**Supplementary Materials**


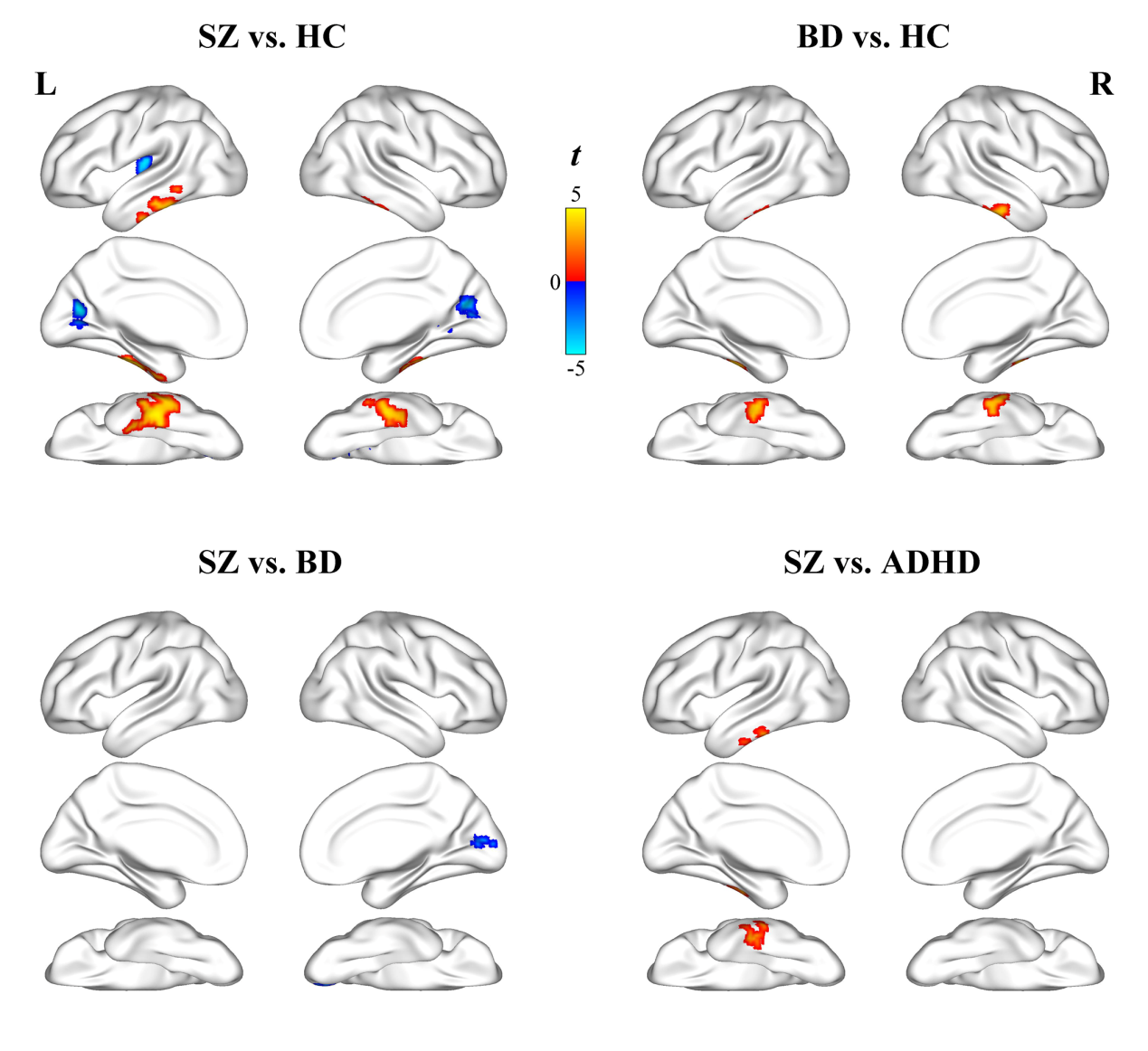


**Fig. S1.** Voxel-wise group comparison results of functional stability derived from a combination of window size = 50 s and sliding step = 4 s. Abbreviations: HC, healthy controls; SZ, schizophrenia; BD, bipolar disorder; ADHD, attention deficit/hyperactivity disorder; L, left; R, right.


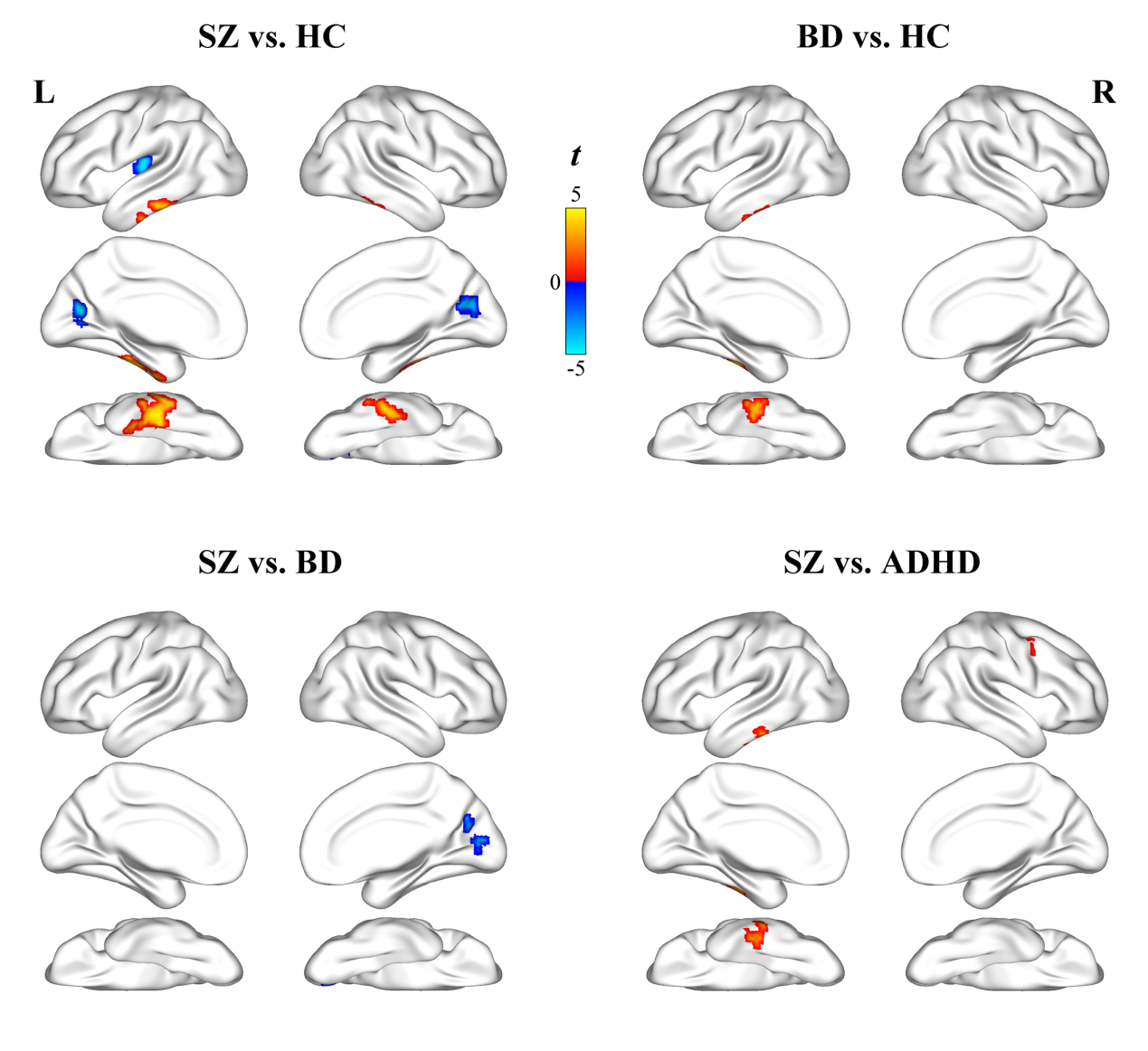


**Fig. S2.** Voxel-wise group comparison results of functional stability derived from a combination of window size = 80 s and sliding step = 4 s. Abbreviations: HC, healthy controls; SZ, schizophrenia; BD, bipolar disorder; ADHD, attention deficit/hyperactivity disorder; L, left; R, right.


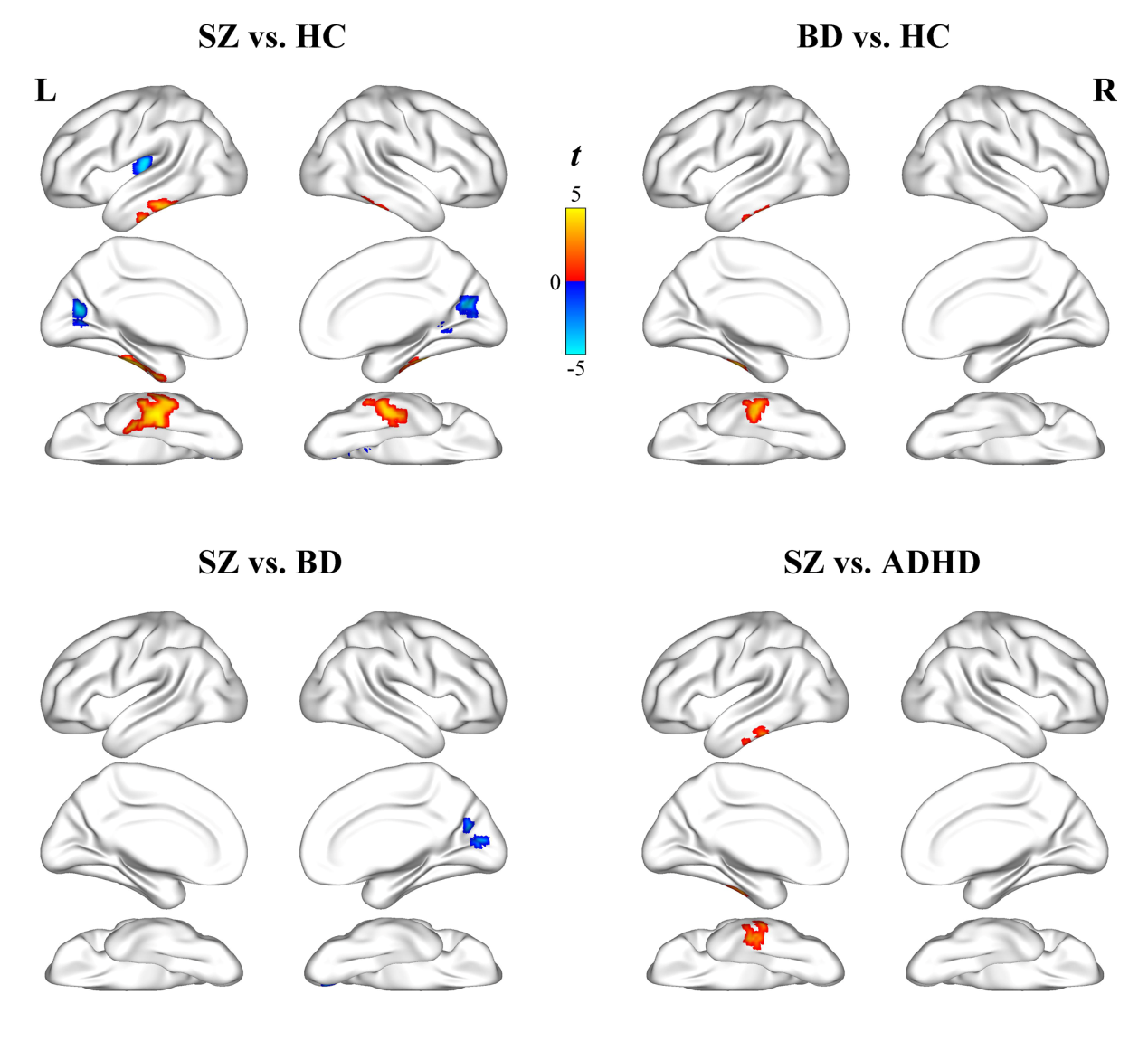


**Fig. S3.** Voxel-wise group comparison results of functional stability derived from a combination of window size = 64 s and sliding step = 2 s. Abbreviations: HC, healthy controls; SZ, schizophrenia; BD, bipolar disorder; ADHD, attention deficit/hyperactivity disorder; L, left; R, right.


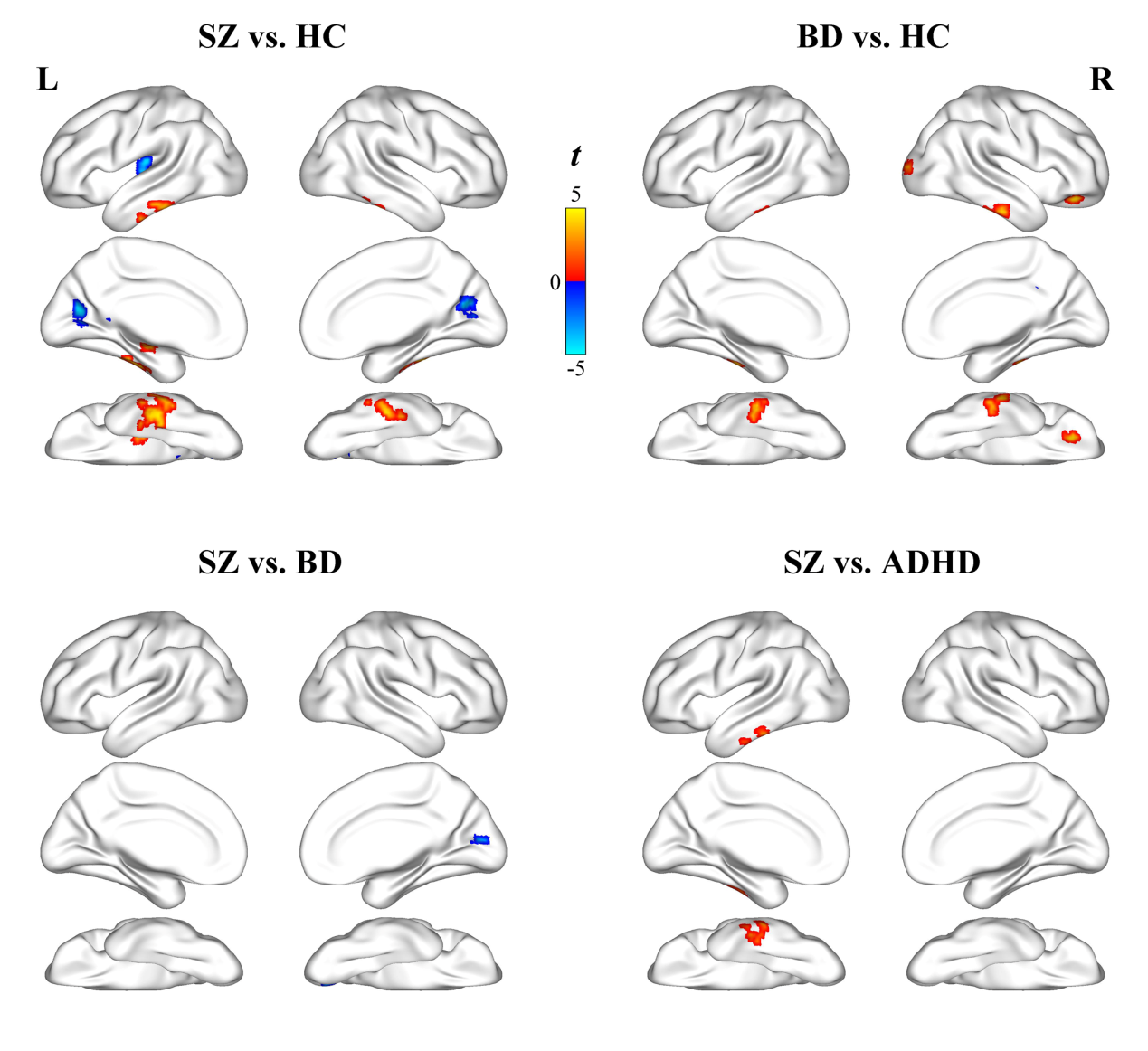


**Fig. S4.** Voxel-wise group comparison results using functional stability maps smoothed by a 4 mm FWHM Gaussian kernel. Abbreviations: FWHM, full-width at half maximum; HC, healthy controls; SZ, schizophrenia; BD, bipolar disorder; ADHD, attention deficit/hyperactivity disorder; L, left; R, right.


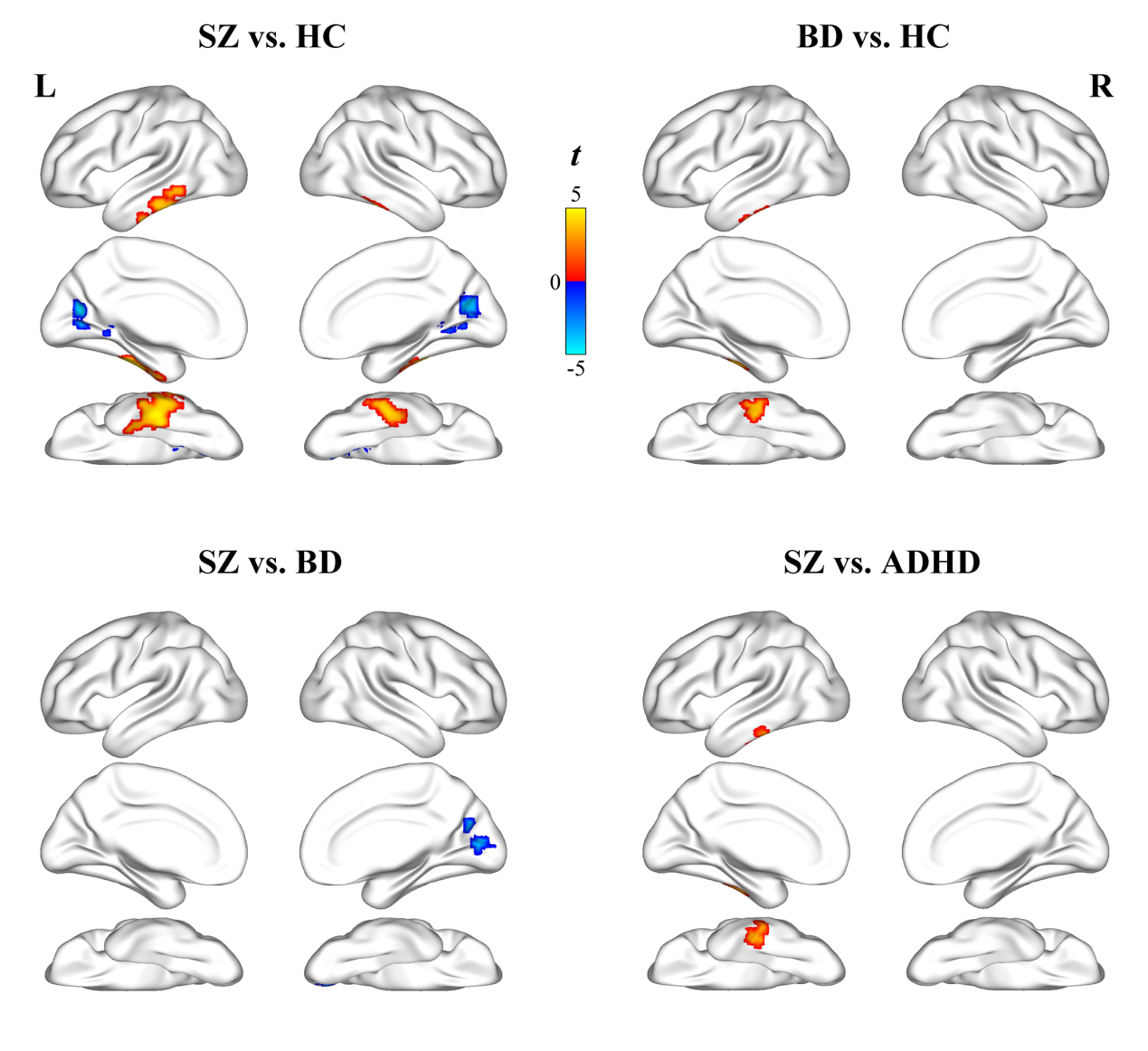


**Fig. S5.** Voxel-wise group comparison results using functional stability maps smoothed by an 8 mm FWHM Gaussian kernel. Abbreviations: FWHM, full-width at half maximum; HC, healthy controls; SZ, schizophrenia; BD, bipolar disorder; ADHD, attention deficit/hyperactivity disorder; L, left; R, right.


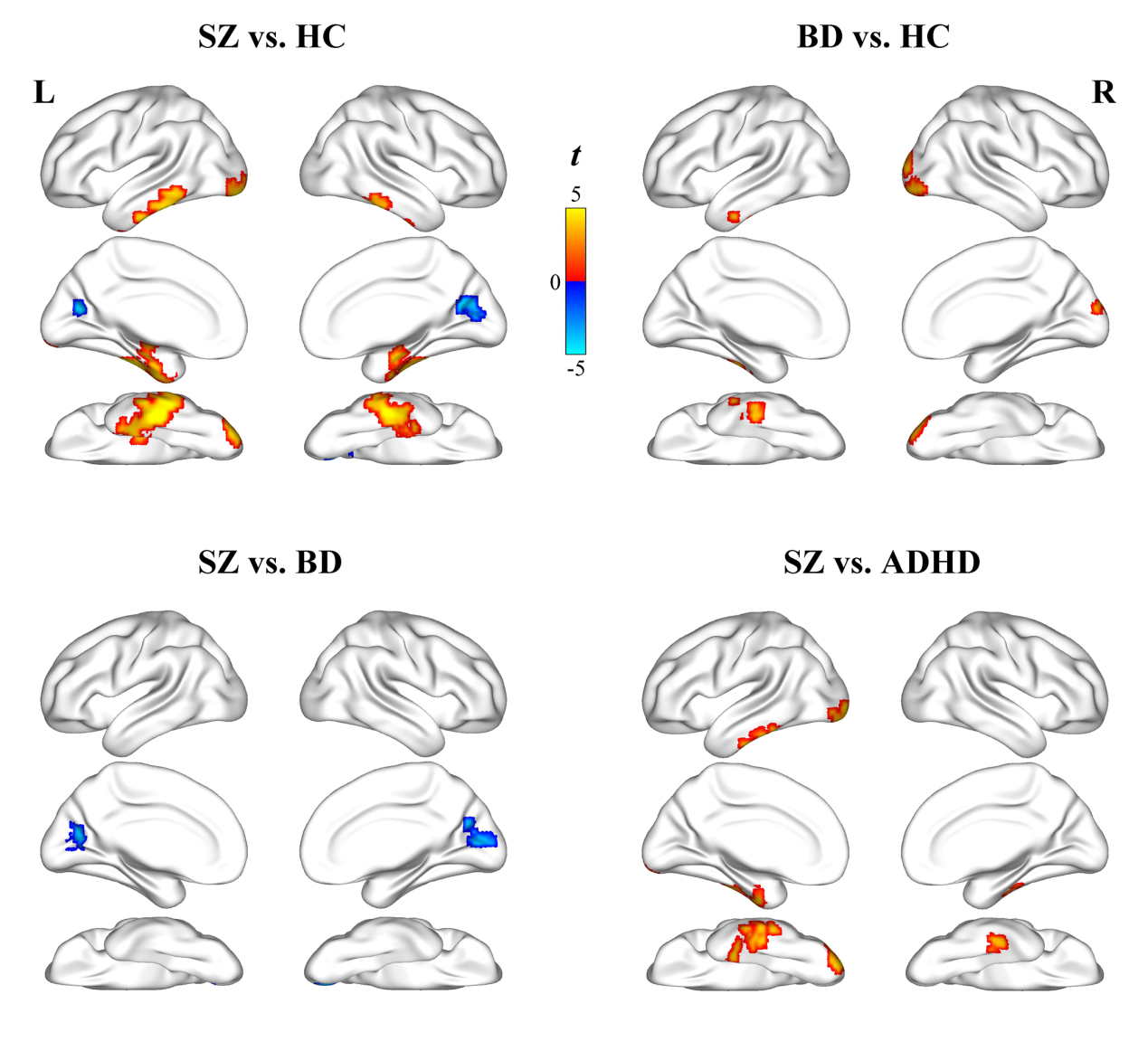


**Fig. S6.** Voxel-wise group comparison results of functional stability using fMRI data with GSR. Abbreviations: HC, healthy controls; SZ, schizophrenia; BD, bipolar disorder; ADHD, attention deficit/hyperactivity disorder; L, left; R, right; fMRI, functional magnetic resonance imaging; GSR, global signal regression.


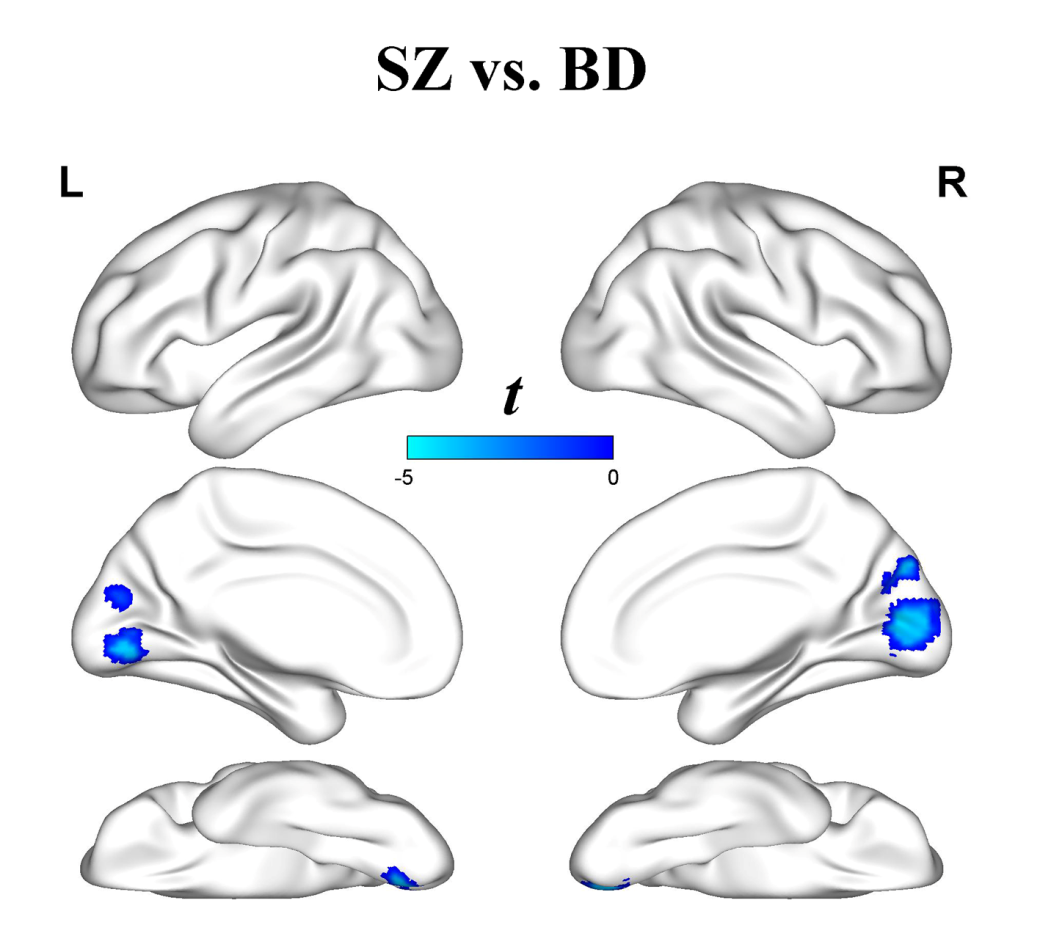


**Fig. S7.** Group comparison results of functional stability using a voxel-to-network approach. Abbreviations: SZ, schizophrenia; BD, bipolar disorder; L, left; R, right.


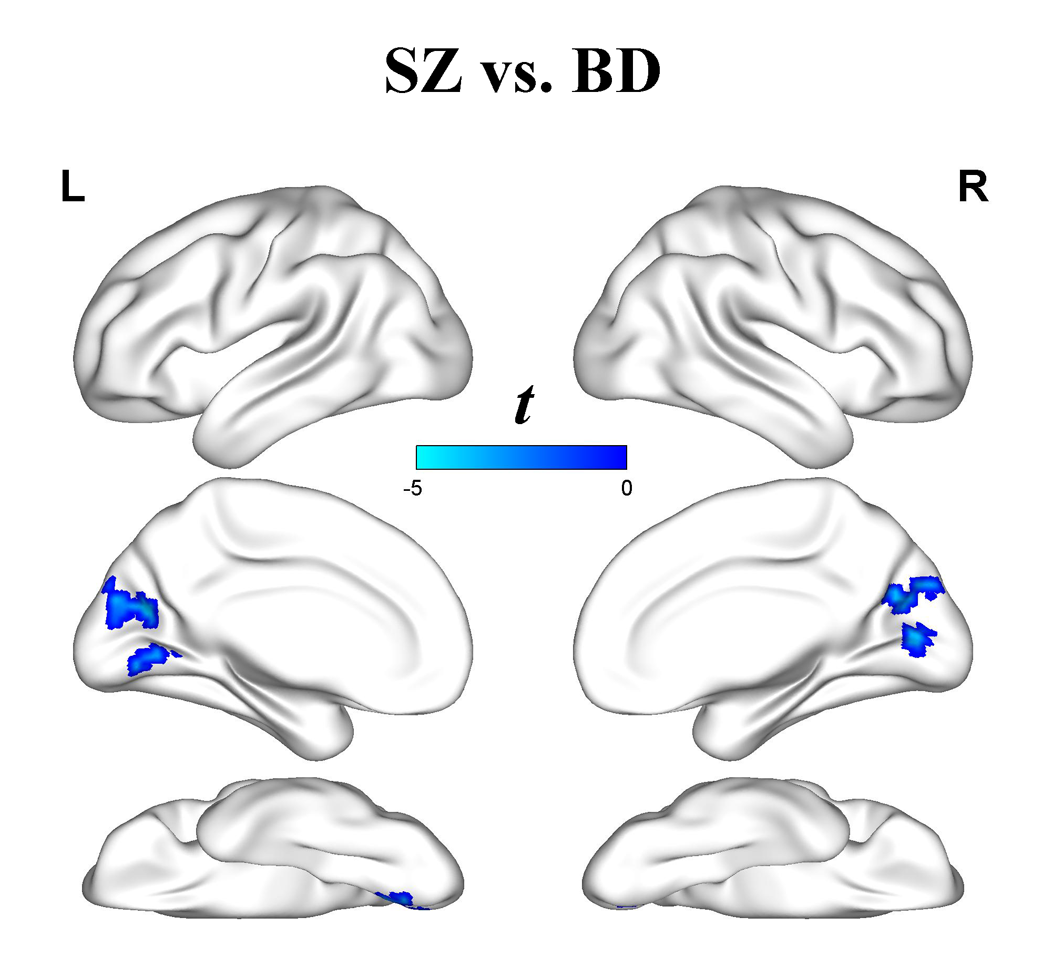


**Fig. S8.** Group comparison results of functional stability derived from smoothed fMRI data. Abbreviations: SZ, schizophrenia; BD, bipolar disorder; L, left; R, right; fMRI, functional magnetic resonance imaging.**Table S1.** Correlations between functional stability and clinical symptoms in individuals with schizophrenia

| Clinical symptoms | B-calcarine sulcus | L-Insula | L-Inferior temporal gyrus | R-Inferior temporal gyrus | L-Amygdala | L-Thalamus |
| --- | --- | --- | --- | --- | --- | --- |
| SAPS hallucinations  SAPS delusions  SAPS bizarre behavior  SAPS thought disorder  SANS affective flattening  SANS alogia  SANS avolition/apathy  SANS anhedonia/asociality  SANS attention  HAMD  YMRS | -0.109 (0.485)  -0.028 (0.860)  -0.011 (0.943)  -0.011 (0.945)  0.260 (0.092)  0.129 (0.410)  -0.061 (0.698)  0.041 (0.796)  < 0.001 (0.999)  0.203 (0.192)  -0.087 (0.587) | 0.311 (0.042)  -0.016 (0.921)  -0.104 (0.507)  -0.014 (0.930)  -0.092 (0.558)  -0.099 (0.527)  0.061 (0.698)  0.193 (0.215)  -0.176 (0.260)  0.166 (0.286)  0.013 (0.937) | -0.012 (0.941)  0.052 (0.741)  -0.031 (0.845)  0.008 (0.962)  -0.252 (0.103)  -0.201 (0.197)  -0.065 (0.681)  -0.361 (0.017)  0.184 (0.237)  -0.056 (0.723)  0.088 (0.574) | -0.009 (0.955)  0.197 (0.206)  -0.150 (0.337)  -0.098 (0.530)  -0.213 (0.171)  -0.221 (0.154)  -0.160 (0.304)  -0.379 (0.012)  0.033 (0.831)  -0.022 (0.890)  -0.033 (0.836) | 0.039 (0.803)  -0.099 (0.528)  -0.003 (0.985)  0.187 (0.230)  0.028 (0.861)  0.088 (0.574)  0.155 (0.320)  0.078 (0.621)  0.033 (0.835)  -0.008 (0.959)  -0.030 (0.850) | 0.247 (0.111)  0.126 (0.422)  0.035 (0.824)  0.094 (0.549)  0.033 (0.836)  0.021 (0.891)  -0.004 (0.980)  0.100 (0.525)  0.013 (0.932)  0.170 (0.275)  0.036 (0.817) |

The data are shown as the partial correlation coefficient (*P* value). Abbreviations: SAPS, Scale for the Assessment of Positive Symptoms; SANS, Scale for the Assessment of Negative Symptoms; HAMD, Hamilton Rating Scale for Depression; YMRS, Young Mania Rating Scale; B, bilateral; L, left; R, right.

**Table S2.** Correlations between functional stability and clinical symptoms in individuals with bipolar disorder

| Clinical symptoms | B-calcarine sulcus | L-Insula | L-Inferior temporal gyrus | R-Inferior temporal gyrus | L-Amygdala | L-Thalamus |
| --- | --- | --- | --- | --- | --- | --- |
| SAPS hallucinations  SAPS delusions  SAPS bizarre behavior  SAPS thought disorder  SANS affective flattening  SANS alogia  SANS avolition/apathy  SANS anhedonia/asociality  SANS attention  HAMD  YMRS | -0.362 (0.023)  0.216 (0.187)  -0.011 (0.947)  0.078 (0.636)  -0.177 (0.282)  -0.206 (0.208)  -0.282 (0.082)  -0.300 (0.063)  0.111 (0.501)  -0.143 (0.386)  -0.006 (0.971) | -0.120 (0.468)  0.231 (0.156)  0.288 (0.076)  0.226 (0.167)  -0.067 (0.683)  -0.236 (0.149)  -0.170 (0.301)  0.012 (0.943)  0.208 (0.205)  -0.055 (0.740)  0.223 (0.173) | 0.168 (0.306)  -0.235 (0.150)  -0.148 (0.370)  -0.098 (0.555)  -0.112 (0.496)  0.362 (0.023)  0.145 (0.380)  0.171 (0.298)  0.067 (0.684)  -0.039 (0.815)  -0.090 (0.586) | -0.038 (0.818)  -0.091 (0.580)  -0.240 (0.142)  -0.054 (0.744)  -0.002 (0.989)  0.322 (0.046)  0.199 (0.225)  0.329 (0.041)  -0.013 (0.939)  0.070 (0.674)  -0.136 (0.410) | 0.074 (0.653)  0.052 (0.752)  0.259 (0.111)  0.284 (0.080)  0.039 (0.812)  -0.026 (0.873)  0.246 (0.131)  0.180 (0.272)  0.159 (0.334)  0.165 (0.315)  0.287 (0.077) | -0.379 (0.017)  -0.120 (0.469)  0.210 (0.200)  -0.067 (0.686)  0.147 (0.371)  0.172 (0.294)  0.397 (0.012)  -0.147 (0.373)  0.257 (0.114)  0.100 (0.546)  -0.123 (0.454) |

The data are shown as the partial correlation coefficient (*P* value). Abbreviations: SAPS, Scale for the Assessment of Positive Symptoms; SANS, Scale for the Assessment of Negative Symptoms; HAMD, Hamilton Rating Scale for Depression; YMRS, Young Mania Rating Scale; B, bilateral; L, left; R, right.

**Table S3.** Correlations between functional stability and clinical symptoms in individuals with attention deficit/hyperactivity disorder

| Clinical symptoms | B-calcarine sulcus | L-Insula | L-Inferior temporal gyrus | R-Inferior temporal gyrus | L-Amygdala | L-Thalamus |
| --- | --- | --- | --- | --- | --- | --- |
| HAMD  YMRS | -0.144 (0.401)  -0.044 (0.798) | 0.288 (0.089)  -0.134 (0.437) | 0.040 (0.815)  -0.021 (0.904) | 0.054 (0.756)  -0.052 (0.762) | 0.127 (0.459)  -0.103 (0.551) | 0.020 (0.910)  0.003 (0.987) |

The data are shown as the partial correlation coefficient (*P* value). Abbreviations: HAMD, Hamilton Rating Scale for Depression; YMRS, Young Mania Rating Scale; B, bilateral; L, left; R, right.

**Table S4.** Correlations between functional stability and clinical symptoms in all psychiatric individuals

| Clinical symptoms | B-calcarine sulcus | L-Insula | L-Inferior temporal gyrus | R-Inferior temporal gyrus | L-Amygdala | L-Thalamus |
| --- | --- | --- | --- | --- | --- | --- |
| SAPS hallucinations  SAPS delusions  SAPS bizarre behavior  SAPS thought disorder  SANS affective flattening  SANS alogia  SANS avolition/apathy  SANS anhedonia/asociality  SANS attention  HAMD  YMRS | -0.276 (0.010)  0.119 (0.276)  -0.058 (0.598)  -0.049 (0.654)  -0.017 (0.880)  -0.159 (0.144)  -0.197 (0.069)  -0.164 (0.132)  -0.116 (0.288)  0.026 (0.809)  -0.025 (0.819) | -0.016 (0.884)  0.168 (0.122)  0.033 (0.762)  0.016 (0.886)  -0.150 (0.168)  -0.238 (0.028)  -0.118 (0.281)  0.014 (0.902)  -0.057 (0.603)  0.029 (0.792)  0.150 (0.169) | 0.065 (0.553)  -0.102 (0.352)  -0.076 (0.487)  -0.026 (0.810)  -0.141 (0.196)  0.009 (0.936)  0.028 (0.796)  -0.126 (0.248)  0.170 (0.118)  -0.070 (0.520)  -0.031 (0.778) | 0.067 (0.539)  0.013 (0.904)  -0.175 (0.106)  -0.073 (0.507)  -0.072 (0.508)  0.015 (0.888)  0.011 (0.919)  -0.045 (0.679)  0.054 (0.619)  0.002 (0.984)  -0.107 (0.327) | 0.087 (0.428)  < 0.001 (0.999)  0.077 (0.480)  0.196 (0.070)  0.077 (0.479)  0.122 (0.263)  0.189 (0.082)  0.095 (0.386)  0.115 (0.293)  0.029 (0.791)  0.089 (0.416) | 0.108 (0.322)  -0.012 (0.913)  0.109 (0.318)  0.044 (0.688)  0.072 (0.513)  0.029 (0.790)  0.161 (0.139)  0.290 (0.007)  0.105 (0.338)  0.099 (0.364)  -0.067 (0.540) |

The data are shown as the partial correlation coefficient (*P* value). Abbreviations: SAPS, Scale for the Assessment of Positive Symptoms; SANS, Scale for the Assessment of Negative Symptoms; HAMD, Hamilton Rating Scale for Depression; YMRS, Young Mania Rating Scale; B, bilateral; L, left; R, right.
